# Evolution of the Early Spliceosomal Complex—From Constitutive to Regulated Splicing

**DOI:** 10.3390/ijms222212444

**Published:** 2021-11-18

**Authors:** Sonia Borao, José Ayté, Stefan Hümmer

**Affiliations:** 1 Oxidative Stress and Cell Cycle Group, Universitat Pompeu Fabra, 08003 Barcelona, Spain; sonia.borao@upf.edu; 2 Translational Molecular Pathology, Vall d’Hebron Research Institute (VHIR), CIBERONC, 08035 Barcelona, Spain

**Keywords:** splicing, spliceosome, E-complex, Prp2, 5′ splicing site, exon–intron junction, fission yeast, U2AF65

## Abstract

Pre-mRNA splicing is a major process in the regulated expression of genes in eukaryotes, and alternative splicing is used to generate different proteins from the same coding gene. Splicing is a catalytic process that removes introns and ligates exons to create the RNA sequence that codifies the final protein. While this is achieved in an autocatalytic process in ancestral group II introns in prokaryotes, the spliceosome has evolved during eukaryogenesis to assist in this process and to finally provide the opportunity for intron-specific splicing. In the early stage of splicing, the RNA 5′ and 3′ splice sites must be brought within proximity to correctly assemble the active spliceosome and perform the excision and ligation reactions. The assembly of this first complex, termed E-complex, is currently the least understood process. We focused in this review on the formation of the E-complex and compared its composition and function in three different organisms. We highlight the common ancestral mechanisms in *S. cerevisiae*, *S. pombe*, and mammals and conclude with a unifying model for intron definition in constitutive and regulated co-transcriptional splicing.

## 1. Introduction

Splicing of mRNA precursors is an essential part of regulated gene expression. The process consists in the excision of the introns (non-coding sequences) from the precursor mRNA (pre-mRNA), and results in the ligation of the coding sequences (exons), forming the mature mRNA. This is achieved by two consecutive trans-esterification reactions, which need to occur at nucleotide precision to avoid frame shifting with adverse consequences on the protein coding potential of the mRNA. To achieve this accuracy, sequence specific cis-acting elements on the pre-mRNA define the exon intron junctions. Evidence indicates that splicing has evolved during eukaryogenesis from self-splicing group II introns of prokaryotes together with the spliceosome acting in trans, to catalyze the splicing reaction [1,2]. Two types of spliceosomes are present across eukaryotes, namely the major and the minor spliceosome. Each spliceosome splices its own type of introns, the U2-type introns for the major spliceosome and the U12-type introns for the minor counterpart. The core mechanism of U2-type splicing is conserved from yeast to higher eukaryotes, as is the spliceosome [3]. However, U12 introns (and the associated snRNAs) are absent from the yeasts *Saccharomyces cerevisiae* and *Schizosaccharomyces pombe* [4]. In this review, we will focus on the major spliceosome and U2-type introns and compare introns and the splicing machinery between *S. cerevisae*, *S. pombe*, and humans to highlight common ancestor mechanisms and how their increase in complexity over evolution might enable the transition from constitutive to regulated and alternative splicing.

## 2. The Spliceosome

The spliceosome is a multi-component machine composed of five small nuclear RNAs (snRNAs) pre-assembled with proteins into small ribonucleoproteins (snRNPs) and hundreds of additional proteins. The five different snRNPs are called U1, U2, U4, U5, and U6 [5,6,7]. The assembly of the spliceosome takes place through multiple dynamic interactions that leads to the formation of different intermediate complexes: E (ATP independent), A, B, and C (ATP dependent) [8,9,10].

In the first step of spliceosome assembly, the exon intron junctions are defined by recognition and interaction with the cis-acting elements on the pre-mRNA termed the 5′ and 3′ splice sites (5′ ss and 3′ ss) [11]. The formation of the first spliceosomal complex (E-complex) is initiated by U1 snRNP interaction with the 5′ ss and the cooperative recognition of the 3′ ss by SF1 (Splicing Factor 1) and U2AF (U2 snRNP auxiliary factor). The A-complex is formed when U2 snRNP displaces SF1. Next, U4/U5-U6 tri-snRNP binding to 5′ ss results in the formation of the pre-catalytic B-complex and rearrangements of RNA-RNA and RNA-protein interactions lead to the catalytic active spliceosome. After completion of the first trans-esterification reaction, the C-complex is formed, which carries out the second reaction of splicing [11,12].

## 3. The Evolution of the Spliceosome

The core machinery of the U2-type spliceosome is highly conserved across eukaryotes [13,14]. Comparison of the spliceosomal components between *S. pombe*, *S. cerevisiae*, and humans confirmed this conservation [3]. There are, however, some factors that are present in fission yeast and humans that appear to be absent in budding yeast and several factors are present in humans that do not exist in either yeast. As the basic splicing mechanism is functional in all three organisms, the increase in the complexity of the spliceosome is thought to contribute to the evolution from mainly constitutive splicing in budding yeast to a highly regulated and alternative splicing in humans.

Due to the evolutionary conservation of the splicing machinery, studies in the fission and budding yeasts have been fundamental for the discovery of spliceosomal components and for the dissection of basic mechanisms of splicing [3,15,16,17,18,19,20,21,22]. There are, however, major differences in the splicing machinery between the two yeast species. While phylogenetic studies revealed that many proteins of the splicing machinery are well conserved, about 40% of the fission yeast splicing factors are more similar to the human proteins than to the budding yeast proteins [3,19]. Most of these factors are described to play a role in the recognition of the 3′ ss. [3,23]. In this line, members of the family of serine/arginine (SR)-rich proteins, which have been shown to interact with proteins that recognize the 3′ splice site, are found in both fission yeast and humans but are absent in budding yeast. The higher degree of conservation of transacting splicing factors parallels the high degree of degeneracy of splice site sequences in *S. pombe*, which closely reflects the observation in human transcripts [13]. For this and other reasons detailed below, it is considered that *S. pombe* represents an evolutionary intermediate between the constitutive mechanism of splicing in *S. cerevisiae* and the dynamically regulated process of splicing in humans, which allows alternative splicing of the same pre-mRNA into different mRNAs.

## 4. Regulated and Alternative Splicing

Alternative splicing can produce a variety of mRNAs from a single gene in which some of the coding exons might be either excluded or only being partially present. Fundamental for this process is the regulation of splicing in an intron specific manner. Even though RNA splicing was originally discovered in the 1970s, the significance of alternative splicing for humans could not be thoroughly appreciated until the Human Genome Project determined that there are approximately 22,000 protein-coding genes that translate to over 90,000 different proteins [24].

**Figure 1:**
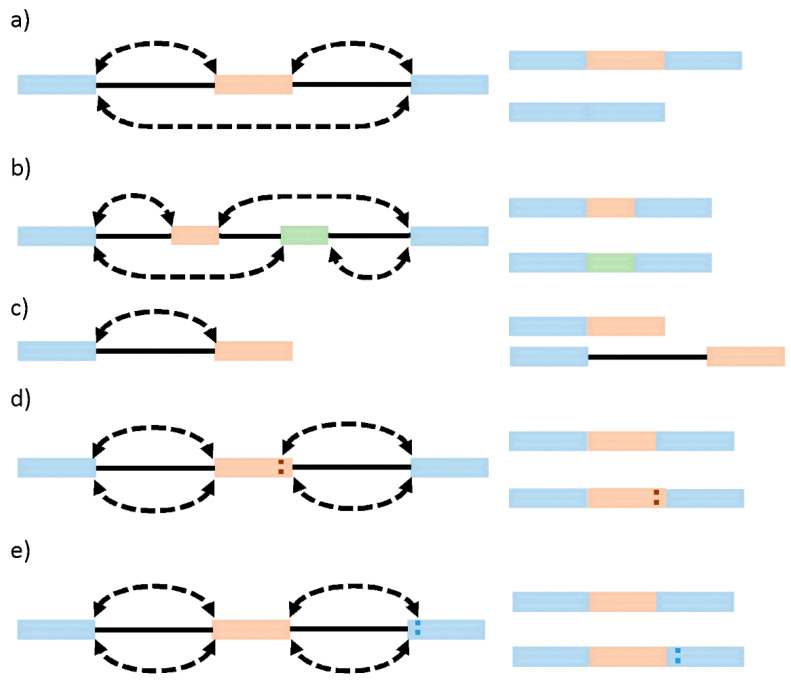
Modes of alternative splicing. (**a**) Exon skipping. (**b**) Mutually exclusive exons. (**c**) Intron retention. (**d**) Alternative 5′ ss. (**e**) Alternative 3′ ss. Lines indicate introns; blue bars indicate constitutive exons; green and orange bars indicate alternative spliced exons; dotted arrows indicate splicing events; dotted vertical lines indicate alternative splice sites.

Systematic analyses of sequencing and microarray data have so far revealed that besides constitutive splicing, six main types of alternative splicing exist: exon skipping (cassette alternative exon), intron retention, alternative 3′ or 5′ splice site usage, and mutually exclusive exons [25] (Figure 1). While in vertebrates and invertebrates, exon skipping is the most prevalent pattern (~30%) of alternative splicing, in lower metazoans, it is intron retention [26]. Generally, intron retention has been associated with weaker splice sites, short intron length, and the regulation of cis-regulatory elements [27]. Mutually exclusive exons represent a rare subtype of an alternative splicing event that generates alternative isoforms by retaining only one exon of a cluster of neighboring exons in the mature transcript [28]. In this case, two (or more) splicing events are no longer independent, instead executed or disabled in a coordinated manner, which requires an intensive regulatory mechanism [29]. Alternative 3′ or 5′ splice sites are often found in close proximity and with high symmetry levels to the constitutive splice site, allowing the conservation of the open reading frame [30]. It is currently estimated that alternative 3′ ss and/or 5′ ss exons account for about 18% and 8% of alternative transcripts in higher eukaryotes. Those numbers might underestimate the extent of alternative 3′ ss and 5′ ss usage, as the close proximity to the constitutive sides might require full-length RNA sequencing techniques to provide a sufficient resolution.

## 5. Coupling Splicing to Transcription

Transcription of pre-mRNAs by RNA polymerase II (Pol II) and their splicing by the spliceosome are essential steps in gene expression. While both processes are often studied separate from each other in vitro, there is substantial evidence that they are coordinated with each other in a cellular context. Co-transcriptional splicing enhances the efficiency and accuracy of pre-mRNA processing and might explain why splicing is at least ten times faster in vivo than in vitro [31,32,33,34]. The first evidence for co-transcriptional splicing came from electron micrographs of *Drosophila melanogaster* embryonic transcription units, which indicated spliceosome assembly and subsequent intron looping, while the transcript remains tethered to the chromatin template [35,36]. Over the years, an increasing body of evidence supports the notion that transcription and splicing are physically and functionally coupled. In fact, the rate of Pol II elongation can affect selection of splice sites in pre-mRNA [37,38,39,40] leading to alternative splicing and different mRNA isoforms [31,41,42]. Moreover, Pol II can recruit splicing factors via its carboxyl-terminal domain (CTD) [43,44,45,46,47,48,49,50]. The CTD consists of 52 tandem repeats of the heptapeptide YSPTSPS in mammals and 26 tandem repeats in yeast [51], which act as a special platform to recruit different factors to the nascent transcripts via dynamic phosphorylation of several residues. Individual serine residues in the tandem repeat have been shown to be phosphorylated by different kinases and affect binding to different factors involved in transcriptional regulation [52]. The phosphorylation of serine 7 in particular has been found to facilitate elongation and splicing [53]. Moreover, the chromatin environment also influence transcription rates and thereby affects mRNA splicing. The histone 2A variant H2A.Z, for example, promotes efficient pre-mRNA splicing of introns with non-consensus splice sites in both budding and fission yeast [54,55].

Currently, two models have been suggested to explain the co-transcriptional regulation of alternative splicing. The recruitment model, mainly based on the CTD, explains the direct physical coupling of the transcription and splicing apparatus. A key molecule in this physical linkage is PRP40, a member of the U1 complex that can directly bind to the phosphorylated CTD of Pol II [56]. Alternatively, the kinetic model provides an additional model and is based on the different elongation rates of Pol II, which in turn determines the availability of splices sites [57,58]. Despite all the evidence gathered above, additional linkage between Pol II and the splicing machinery must exist, as the CTD is not sufficient to enhance the efficiency of pre-mRNA splicing in the context of a different polymerase [50].

## 6. Co-Transcriptional Splicing of Long Introns and Intron Looping

The spliceosomal E-complex brings the two ends of the intron in close proximity to initiate the process of splicing and looping of the intronic sequence is a prerequisite. While these intron loops have already been described in early electron micrographs of *Drosophila melanogaster* embryonic transcription units [35,36], the underlying molecular mechanisms of intron looping have been just recently started to be explored in more detail. The cryoEM structure of a mammalian transcribing Pol II-U1 snRNP complex has been recently resolved [59]. The structure reveals that Pol II and U1 snRNP interact directly without involvement of the CTD of Pol II. The interaction instead is mediated by the protrusion domain in Pol II subunit RPB2 and the zinc finger domain in subunit RPB12, which contact with the RRM domain of the conserved and functionally essential subunit U1-70k from the U1 snRNP.

Using a CRISPR-based approach to halt RNA polymerase II transcription in the middle of introns, it has been demonstrated that the nascent 5′ splice site base pairs with a U1 snRNA that is tethered to RNA polymerase II during intron synthesis [60]. This mechanism relies on the strength of the 5′ ss–snU1 RNA and enables the co-transcriptional assembly of the E-complex by ensuring proximity of the U1 snRNP at the 5′ ss and the SF1-U2AF complex at the 3′ ss. In this way, not only short introns—as previously thought—but also long introns might be rapidly spliced co-transcriptionally if the 5′ ss-snU1 and the snU1-Pol II interaction is sufficiently strong to ensure looping of the intron, forcing the proximity of the intron borders.

## 7. The Evolution of Intron Architecture and Intron-Exon Structures

The intron density greatly differs between *S. cerevisiae*, *S. pombe*, and humans. Even though they have a similar number of coding genes (about 5000), *S. cerevisae* only contains about 300 introns, in contrast to almost 5000 introns in *S. pombe*. In the latter case, almost half of its genes contain introns (~45%), and many of them have more than one [61,62]. In comparison, the human genome contains around 20,000 protein-coding genes, and most of them have multiple introns [24,63,64]). The intron architecture also differs significantly between the two yeast species. In *S. pombe*, the introns have an average length of less than 100 nt. The mean intron length for *S. cerevisiae* is significantly larger and shows a bimodal pattern, with approximately 25% of the *S. cerevisiae* introns larger than 400 nt [62]. However, considering the total number of introns, *S. pombe* also has more than 75 introns with a length of more than 400 nt. Introns in humans are, on average, much longer than in yeasts, albeit very heterogeneous in length. Most introns in human pre-mRNAs fall either within a narrow peak under 100 nt or in a broad distribution peaking around several thousand nucleotides and extending to over a million nucleotides [65,66].

## 8. Definition of the Exon Intron Boarders by the Spliceosome

**Figure 2:**
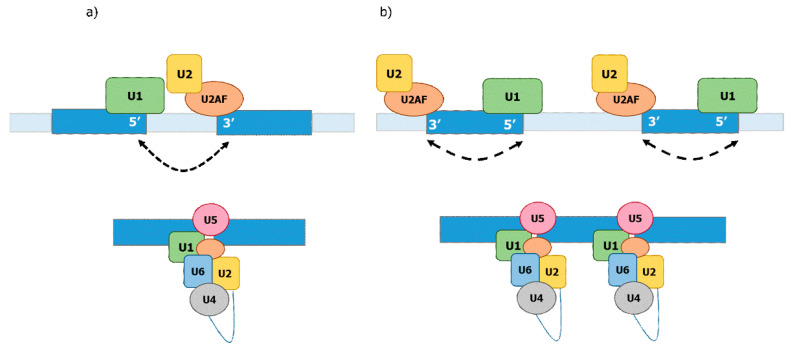
Definition of the exon–intron junctions. (**a**) Intron definition. (**b**) Exon definition. Darker blue bars indicate exons; lighter blue bars indicate introns; blue line indicate intron lariat.

Currently, two mechanisms are described for the definition of the exon–intron junctions: exon- and intron definition (Figure 2). Intron definition is thought to be prevalent in the case of smaller introns relative to larger exons and is considered to be the ancient mechanism of splicing [13]. Exon definition is primarily thought to take place in the case of relatively long introns and shorter exons wherein the splicing machinery establishes over the exons [13,67,68,69,70]. Evidence for these two mechanisms has been derived from analyses of interactions between pre-mRNAs and various splicing factors [13,69,71]. The exon definition mechanism involves SR proteins binding to exonic splicing enhancers (ESE) and recruiting U1 to the downstream donor splicing signal and the splicing factor U2AF to the upstream acceptor splicing signal. The U2AF factor then recruits U2 to the branch site. Therefore, when the SR proteins bind the ESEs, they promote the formation of a “cross-exon” recognition complex by placing the basal splicing machinery at the splice sites flanking the same exon. The intron definition mechanism requires binding of U1 to the upstream donor splice site and binding of U2AF/U2 to the downstream acceptor splice signal and branch site of the same intron. Therefore, intron definition selects pairs of splice sites located on both ends of the same intron [69,72] (Figure 2). The efficiency of splicing under the exon definition depends on the length of exons but is not affected by the length of introns, while the efficiency of splicing under intron definition depends on the length of introns but not on that of exons [13,68,69,70,71,73].

Based on the exon–intron characteristics in the different organisms, exon definition has been thought to be prevalent for splicing of introns in higher eukaryotes while intron definition is predominant in lower eukaryotes such as yeast. This view is challenged by the fact that there is a substantial portion of introns in humans that have a similar length than those in yeast. Moreover, the difficulty to join the 5′ ss and 3′ ss in long human introns has been used as an argument for the exon definition model. However, recent evidence indicates that the 5′ ss may stay attached to Pol II during transcriptional elongation, which would thereby enable the intron definition model for long introns, as discussed above [13,59]. Remarkably, studies on the mechanism of splice site selection have been largely carried out in vitro, in which splicing is uncoupled from transcription. Taking all of this into account, it might be important in future to revise the mechanisms of splice site selection, taking into account the intron length and time required for transcription of a given intron. A plausible consensus might be that fast transcribed introns could rely on a co-transcriptional intron definition mechanism, while slow transcribed introns, which are not able to assemble the pre-spliceosomal complex during transcription, rely on the exon definition.

## 9. The Spliceosomal E-Complex

Crucial for the definition of the exon–intron junctions is the early spliceosomal complex (E complex), also called commitment complex (CC) in yeast. This minimal complex consists of the U1-snRNP, SF1, and U2AF [74,75,76,77] and is sufficient to recognize all intron defining *cis* elements. Base pairing between the 5′ ss and the 5′-end of U1 snRNA defines the start of the intron [78,79,80]. The BP site is directly recognized by the conserved pre-mRNA splicing factor SF1 [81]. Recognition of the 3′ ss is achieved by the U2AF heterodimer, composed of the 65-kDa subunit (U2AF65) and the 35-kDa subunit (U2AF35). Current models suggest that U2AF65 recognizes the poly-pyrimidine (Py) tract and U2AF35 recognizes the AG dinucleotide at the 3′ ss [82,83,84,85,86]. Moreover, the binding of U2AF65 to SF1 increases the affinity of SF1 to the pre-mRNA BP site, and this cooperative interaction enables the full recognition of functional 3′ ss and BP at the end of the intron [87,88,89]). Once the pre-spliceosomal complex is assembled, SF1 is displaced from U2AF65 and replaced by the U2 snRNP protein SF3b155/SAP155 [90,91].

## 10. Degeneration of Splice Sites I—5′ ss and snU1

**Figure 3:**
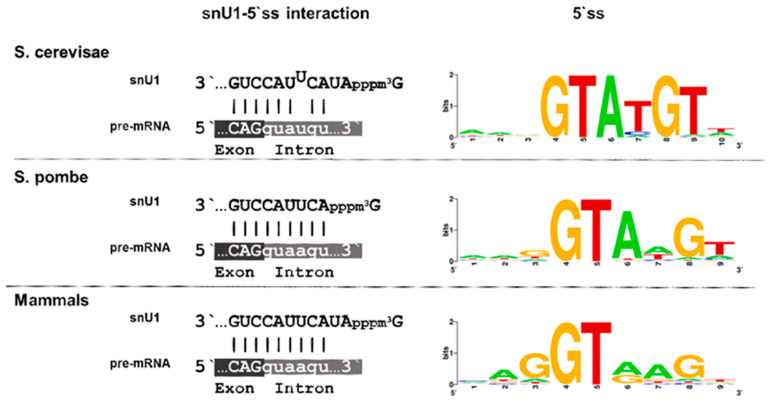
snU1-5′ ss interaction. Nucleotide based interactions of snU1 with the 5′ ss (**left**) and consensus sequence of the 5′ ss (**right**) in *S. cerevisiae*, *S. pombe*, and mammals. Sequence logos were generated by WebLogo (Version 2.8.2) and sequences of the 5′ ss were derived from: *S. cerevisiae* [13], *S. pombe* [98], and mammals [99].

Splice site signals are present in *S. pombe* and *S. cerevisae*, but the degree of conservation largely differs among them. In general, the splicing site signals in *S. cerevisae* do not differ from the consensus type, while they are degenerated in *S. pombe* and humans. The primary function of the 5′ splice site is to ensure the interaction with snU1 through base pairing. The U1 snRNP is the first spliceosomal building block to engage with nascent pre-mRNA, and in humans, it consists of U1 snRNA, seven Sm proteins, and three U1-specific proteins, U1-70k, U1A, and U1C [92,93,94]. These proteins are assembled onto the snU1 RNA, and early studies have revealed a high structural similarity between human and *S. pombe* snU1, while several structures with homology to the conserved stem-loops are less conserved in *S. cerevisae* [95]. The single stranded sequence of snU1 at the 3′ end, which recognizes the 5′ ss through base-pairing, is identical in all three organisms, and only the two residues following the cap-structure are absent in *S. pombe* (Figure 3). This, however, does not seem to affect recognition of the 5′ ss, as these two residues are not implicated in the complementary base pairing, either in *S. cerevisae* or in mammals. Strikingly, though, there are differences in the 5′ ss region of the introns in the respective organisms. The 5′ ss is highly similar in most introns of *S. cerevisiae* and is defined by the six conserved intronic nucleotides GUAUGU. This sequence allows base pairing with five of the snU1 nucleotides and a programmed mismatch at position 4 (Figure 3). Not only base pairing between snU1 and the 5′ ss but also the mismatch at position 4 are required for efficient splicing in *S. cerevisiae*, as a perfect hybrid results in a decrease of splicing efficiency [96,97]. The preceding exonic sequence is not conserved in *S. cerevisiae*. Thus, the 5′ ss in *S. cerevisiae* presents a rigid intronic sequence with a defined interaction strength with snU1. In striking contrast to this, only the GU dinucleotide at the beginning of the intron is conserved in *S. pombe* and humans, and all other nucleotides can be found in the remaining positions.

Importantly, the last three nucleotides of the preceding exon also contribute to the binding affinity between the 5′ ss and snU1. This feature is frequently overseen in many bioinformatic analyses as most of the available intron sequences do not contain this information. Our recent analysis of the 5′ ss affinity to snU1 in *S. pombe* has demonstrated that the last nucleotide of the preceding exon plays a crucial role in defining the interaction strength [98]. Thus, the degeneration of the 5′ ss combined with its extension to the exonic region allows a broad range of interaction strength between the snU1-RNP and the intron and is therefore thought to constitute the basics for intron-specific splicing efficiencies. This allows to hypothesize that the regulation of splicing and alternative splicing might have emerged from the accumulation of mutations at the 5′ ss region, leading to a suboptimal recognition by snU1 [13,100].

## 11. Degeneration of Splice Sites II—Branchpoint Binding by SF1

**Figure 4:**
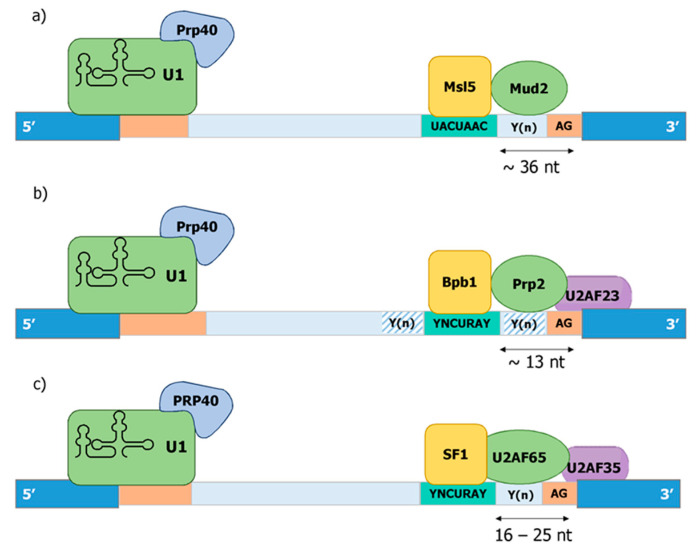
The E-complex in *S. cerevisae*, *S. pombe*, and human. Cis- and trans-elements within the exon and intron in (**a**) budding yeast, (**b**) fission yeast, and (**c**) humans. Distance between BP and AG dinucleotide is represented by arrows.

The 3′ ss determines the end of an intron and is first recognized by SF1/U2AF in the E-complex in the absence of an snRNP. Only later is SF1 replaced by SF3b and snU2. Three cis-elements of an intron—the branchpoint sequence, the Py tract, and the terminal AG dinucleotide—are thought to be involved in defining a functional 3′ ss (Figure 4).

The branch point sequence provides the acceptor nucleotide for the first transesterification reaction with the donor nucleotide at position 1 of the intron. As with the 5′ ss, the branchpoint sequence is highly conserved in budding yeast (UACUAAC). On the contrary, intron lariat sequencing in human and fission yeast have revealed highly degenerated branchpoint sequences with a minimal consensus YURAY (Y = pyrimidine; R = purine) in fission yeast [101,102,103]) and YUNAY (Y = pyrimidine; N = nucleotide(any)) in humans [104]. In all three organisms, the branch point sequence is first recognized by the respective homologues of the human SF1 (Bpb1 in *S. pombe* and Msl5 in *S. cerevisiae*) (Figure 4). In line with this, the optimal binding motif of human SF1 was defined as ACUNAC by RNA crosslinking and immunoprecipitation (CLIP), which resembles the branchpoint sequence [105]. Members of the SF1 family of proteins are characterized by the presence of two types of RNA binding motifs at the N-terminus: a K homology/Quaking 2 (KH/QUA2) domain and one or two zinc knuckle motifs. Among those motifs, KH domain has been shown to be necessary and sufficient for branchpoint sequence binding in yeast [106]. The conserved U2AF-homology Ligand Motif (ULM) at the very N-terminus of SF1 interacts with the U2AF-homology Motif (UHM) of U2AF65 [107]. While SF1 is an essential gene in all three organisms [108,109,110], the requirement for spliceosome assembly and splicing activity appears to be context dependent. In *S. cerevisiae*, mutants of SF1 blocked the formation of the early spliceosomal complex, but splicing activity in in vitro assay was not affected. However, in vivo defects in the splicing of reporter with a weakened 5′ splice site and/or in the branchpoint sequence were observed [111]. Like in *S. cerevisiae*, depletion of SF1 from splicing competent nuclear HeLa cell extract eventually allows spliceosome assembly or splicing, and a kinetic role in splicing was therefore proposed [112]. However, SF1 silencing by siRNA affected splicing of certain but not all transcripts [105,113]. A mutation in SF1 from *S. pombe* causes exon skipping [109]. Thus, SF1 appears to play a conditional role in splicing, and we will describe below how this may impact constitutive and regulated splicing in a co-transcriptional manner.

## 12. The U2AF Heterodimer: Beyond Py Tract Binding—A Physical Link between the Branchpoint and 3′ ss

The U2AF complex has two main functions: defining the functional 3′ ss in the E-complex and assisting in the incorporation of the U2-snRNP in the consecutive step of spliceosome assembly. These different functions are achieved by interaction with different binding partners, which are SF1 in the E-complex and SF3b in the A-complex. We will focus here on the role of the SF1-U2AF complex in defining a functional 3′ ss.

Mammalian U2AF is a heterodimer, composed of the large and small subunit, termed U2AF65 and U2AF35, respectively [83]. The U2AF heterodimer is formed through the interaction of the UHM (U2AF-homology motif) domain in U2AF35 with UHM Ligand Motif (ULM) in U2AF65 [114,115]. *S. pombe* contains homologues for both subunits, which are U2AF59/Prp2 and U2AF23/Uaf2 [88]. In striking contrast to this, *S. cerevisiae* only contains Mud2, a distantly related homolog of U2AF65, but does not contain any U2AF35 homolog (Figure 4).

The conserved AG dinucleotide at the end of an intron is recognized by U2AF35 [84,85,86,116,117,118]). U2AF35 is composed of a UHM flanked by two ZnF binding motifs and a carboxy-terminal RS domain [119,120]. The RS domain is absent from U2AF23 in *S. pombe*, yet the gene is essential for life, like its human counterpart [121]. The RS region of U2AF35 has been shown to establish protein–protein interactions with splicing enhancer proteins of the SR family facilitating the recruitment of U2AF65 [118,122,123]. The requirement on the RS domain of U2AF35 in AG-dependent splicing remains unclear, however [124]. Furthermore, functional studies have revealed that albeit being essential for splicing in vivo, U2AF35 appears to have a conditional role of splicing in vitro [124,125,126]. This discrepancy might be linked to a co-transcriptional splicing as detailed below. Although the AG dinucleotide is conserved at the end of all U2-type introns, there is no homolog of U2AF35 in *S. cerevisiae* [127]. Moreover, the AG dinucleotide is nonessential for completing the first step of splicing in *S. cerevisiae* [125,128]. In this case, the tight binding of the Msl5 to the consensus branchpoint sequence might be sufficient to ensure the first trans-esterification.

U2AF65 belongs to the family of SR proteins and contains an arginine- and serine-rich (RS) domain at the N-terminus, followed by a U2AF-homology ligand motif (ULM), two RNA recognition motifs (RRMs), and a C-terminal U2AF homology motif (UHM) [107,129,130]. While the sequence of U2AF65 is highly conserved from fission yeast to humans, the UHM is the only recognizable portion present in the budding yeast protein Mud2 [87,108,125,127]). This, however, allows the UHM–UHL-based interaction between U2AF65 and SF1 in all organisms. The interaction with the UHM of U2AF35 is mediated by the ULM of U2AF65 in *S. pombe* and humans. Functional analysis also revealed that while U2AF65 in mammals and U2AF59/Prp2 in *S. pombe* are essential for life, Mud2 in *S. cerevisisae* is dispensable [127,131,132].

In mammals, the function of U2AF65 has been described as being carried out by binding to Py tracts located at the 3′ ss between the branchpoint and the terminal AG-dinucleotide [83,133]. Py-tracts are defined as a pyrimidine-rich sequence (C, U) which is not interspaced by a purine (A, G), and the strength of this cis-element is defined by the number of uridines in this region. U2AF65 is sufficient for splicing of introns harboring strong Py-tracts in vitro, whereas the entire U2AF heterodimer is required for the splicing of introns with weak Py-tracts and is essential in vivo [124,125,126,134,135]). In *S. cerevisiae*, Py tracts are weakly defined and, like Mud2, dispensable for splicing [127,136]. In *S. pombe*, a strong Py tract is rarely located between the BP and the 3′ ss (20%) but is located either upstream of the BP or is even absent in the majority of introns [62,98]. Moreover, there is no relationship between the Py tract and the requirement on Prp2 for efficient splicing [98,137]. Thus, in fission yeast, the binding of Prp2 to Py tracts appears to have an assisting function and is dispensable for over 30% of the introns since they lack any recognizable Py tract [98].

Remarkably, most of the studies addressing the requirement of Py tracts for U2AF65 binding and subsequent splicing are based on in vitro splicing reactions uncoupled from transcription and with selected pre-mRNAs. Genome-wide bioinformatics analyses have, however, revealed that a large portion of human introns contains only weak PY tracts which are not likely to represent binding sites for U2AF65 [123,138,139]. From an evolutionary perspective, the requirement on U2AF65 for efficient splicing therefore stems from the stable interaction with SF1 and U2AF35 rather than from binding to Py tracts, as is the case in *S. pombe* [98]. Unifying all these observations, it may be proposed that U2AF65 binding to an intron might rely on two mechanisms: direct interaction with the Py tract or driven by the stable interaction with SF1 and U2AF35. Dependent on any given intron, both mechanisms might act alone or in combination [140]. This context dependency, furthermore, enables regulated and alternative splicing of individual introns.

While Mud2 is dispensable for splicing, U2AF65 was long considered to be absolutely essential. Additionally, in *S. pombe*, most of the introns require Prp2 for efficient splicing, but the degree of dependency on Prp2 is intron specific. In extreme cases, introns are either completely dependent on or independent of Prp2 for efficient splicing [98]. In line with this observation, a conditional role of Prp2 in the splicing of introns with unconventional Py tracts has been proposed [137,141]. Current work on the role of U2AF65 in regulated and constitutive splicing has revealed that U2AF has a maximal capacity to recognize 88% of functional 3′ ss in the human genome [142]. Moreover, the existence of a U2AF65-independent pre-spliceosomal E-complex has been reported in humans, implying that this first step of intron assembly might be carried out in some instances in the absence of U2AF65 [75]. The introns not recognized by U2AF65 might be spliced in a similar manner than the ones in *S. cerevisiae*, where Mud2 is not essential [143,144]).

Binding of the SF1-U2AF complex to the pre-mRNA takes place in the region between the branch point and the 3′ ss. While the length of the introns is very different between human and yeast species, the distance between the branch point and the 3′ ss is remarkably conserved between humans and *S. pombe*. The optimal distance between the branch point to the 3′ ss in human introns is 16–25 nt, with a minimal distance of 8 nt [145]. This is quite similar in *S. pombe*, wherein the average distance is 13 nt [62,98]. In contrast to this, the distance between the branch point and the 3′ ss is highly variable and overall larger in *S. cerevisae* with an average of 36 nt [62]. Therefore, the existence of the trimeric SF1-U2AF65-U2AF35 complex correlates with a defined length of the 3′ ss, which allows the simultaneous recognition of the branch point and the terminal AG-dinucleotide. In this regard, the cooperative binding of the trimeric complex to functional 3′ ss in *S. pombe* has been reported [88]. The definition of functional 3′ ss by at least three criteria, the sequence of the branch point, the distance between branch point and terminal AG dinucleotide, and the presence of poly-pryrimidine tracts allows an even higher degree of complexity, which is thought to be key for the development of highly regulated and alternative splicing over the course of evolution.

## 13. PRP40—A Physical Link between Pol II, snU1-RNP, and SF1/U2AF

The family of Prp40-like proteins is characterized by a WW repeat domain in the amino terminus followed by several FF domains. The WW domain was named after two conserved tryptophan (W) residues in a distance of 20–22 amino acids and mediates specific protein–protein interactions with short proline-rich motifs [146]. The FF domain, named after two conserved phenylalanine (F) residues, has a unique structure that differs significantly from other phosphoserine/threonine-binding domains [147]. Only a few proteins contain the characteristic WW-FF domain architecture, and all of those have been implicated in transcription and/or splicing. This has led to the proposal that WW-FF domain-containing proteins may participate in the functional coupling of transcriptional elongation with splicing [148].

Prp40, the founding member of this family, was identified as an essential factor for splicing in *S. cerevisiae*, which genetically and physically associates with the U1 snRNP [149]. Later on, Prp40 was shown to directly interact with Msl5 (SF1) through its WW domains and indirectly with Mud2p [108]. Based on both findings, a model for the cross-intron interaction in the earlier steps of spliceosome complex formation was proposed. In this model, Prp40 helps to define the bridging interaction that links both ends of the intron by interacting simultaneously with Msl5/Mud2 and the U1 snRNP [108] (Figure 4). *S. pombe* contains a homolog of budding yeast Prp40, which is considered to be part of the U1 sRNP [150].

In mammals, three Prp40-like proteins have been described: PRPF40A, PRPF40B, and TCERG1. PRPF40A interacts with SF1 and U2AF65 and is present in the human A and B, but not C, complexes [9]. Immunoprecipitation of PRPF40A from HeLa nuclear also revealed the binding to the U2 snRNP and many proteins involved in the regulation of transcriptional elongation by Pol II [151]. These data are consistent with a role of PRPF40A during the early assembly of the spliceosomal complex.

PRPF40B is not so well characterized. The protein is found to be localized to splicing factor-rich nuclear speckles and associates with SF1 and U2AF65 [152]. Functional assays revealed the implication of PRPF40B in the selection of alternative splice sites of apoptotic genes [152]. Interestingly, PRPF40B-mediated alternative splicing of Fas relies on weak 5′ ss and 3′ ss, which are recognized in the E-complex by the U1 snRNP and SF1/U2AF, respectively [152]. In line with this, RNA sequencing of PRPF40B knock-out cells revealed hundreds of alternative splicing events, particularly of introns with weak 5′ ss [153].

The third member of the Prp40 family, TCERG1, has also been implicated in transcriptional elongation and pre-mRNA splicing [154]. TCERG1 has been found in A and B, but not in C spliceosomal complexes, by mass spectrometry, and the WW domains of TCERG1 interact with the splicing factors SF1 and U2AF65 [155,156,157]. Moreover, several splicing reporters as well as endogenous mRNAs have been shown to depend on TCERG1 for efficient splicing [155,158,159,160,161,162]. However, it appears to be dispensable for splicing in vitro, which may indicate that requirement on TCERG1 for efficient splicing is linked to transcription [155].

## 14. An Evolutionary Derived Model for Co-Transcriptional Formation of the E-Complex

**Figure 5:**
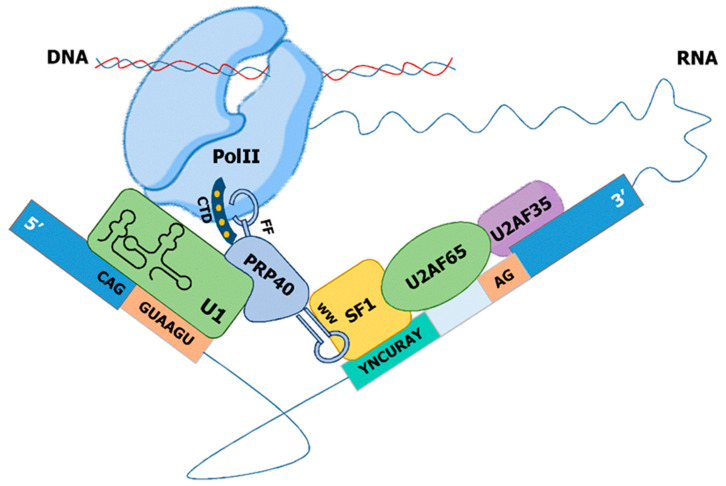
Unified model for co-transcriptional assembly of the E-complex. Co-transcriptional splicing where snRNP U1 subunit U1-70k binds RNA Pol II directly, and Prp40 FF domain binds to the RNA Pol II phosphorylated CTD. Prp40 WW domain binds directly to SF1, bridging 5′ ss and 3′ ss.

The minimal E-complex in *S. cerevisae*, *S. pombe*, and mammals is composed of the snU1-RNP and SF1 physically linked to each other via Prp40. This E-complex, originally termed commitment complex, has the capacity to recognize the 5′ ss and the branchpoint sequence. In the case of consensus splice sites (such as in *S. cerevisiae*), this minimal complex is sufficient to initiate the formation of the catalytically active spliceosome. During evolution, the splice site motifs have degenerated to allow regulated splicing. In the case of *S. pombe*, strong interactions at introns with consensus such as splice sites can still be carried out by the minimal E-complex, but introns with degenerated motifs require the U2AF heterodimer. The major cis-acting element determining the requirement on U2AF for efficient splicing in fission yeast is the strength of the 5′ ss-snU1 interaction [98]. Importantly, the exonic part of the 5′ ss, often not considered in the analysis of intronic *cis*-elements, plays a major role in the interaction between the 5′ ss and snU1. U2AF65 interacts with SF1 and thereby bridges the interaction at the branch side with the recognition of the terminal AG-dinucleotide by U2AF35. This 3′ ss complex is linked via Prp40 to the snU1-RNP at the 5′ ss and enables a highly cooperative recognition of the degenerated splice site motifs across the entire intron. This allows further intron-specific regulation of splicing, as the primary sequence of each intron defines a discrete interaction strength with the tans-acting E-complex. Finally, this complex can be assembled co-transcriptionally as Prp40 and U1-70K interact with Pol II. Thus, a stepwise co-transcriptional assembly of the E-complex through recognition of 5′ ss via snU1 followed by the cooperative recognition of the branch-point and the AG dinucleotide at the 3′ ss via SF1-U2AF can be achieved (Figure 5).

Although there is no direct experimental proof, it is tempting to speculate that snU1 linked to Pol II may scan the pre-mRNA in the 5′–3′ direction as the transcript elongates and eventually binds to the putative 5′ ss if the interaction is sufficiently strong. This scanning-like process can be simulated in silico by determining the interaction strength of the snU1 with the region surrounding the 5′ ss in a sliding window analysis [98]. When recapitulating this process with introns of *S. pombe* that have different requirements on Prp2 for splicing, two features can be observed: (1) for introns with a strong dependency on Prp2, the maximal interaction strength is lower than in Prp2-independent introns; (2) introns with low dependency on Prp2 have a broader entropy valley, indicating that movements of the snU1 RNA in this frame can be tolerated and will not lead instantly to a loss of the interaction with the 5′ ss. Finally, we hypothesize that whether the 3′ ss is recognized by the SF1-U2AF complex, which is linked to the snU1-RNP via Prp40, the formation of the E-complex can be completed at the same speed than the transcript is elongated. This would ultimately allow a fast process of splicing as reported in vivo, which would be at the same time efficient and robust enough to sustain the correct reading frame of the processed transcript.

Based on this model, the exon–intron junctions are established according to the intron definition model. While this was originally thought to be mainly relevant for short introns in lower eukaryotes, this may also account for a large portion of introns in metazoans. Firstly, there is a large fraction of introns in metazoans with a similar size than those in fission yeast. Secondly, it is not the intron length but the speed of transcription of an individual intron that defines how long the U1-snRNP needs to remain attached to Pol-II until the 3′ ss is reached. Fast transcription rates may allow also intron definition of splicing for long introns, while low transcription rates may provoke pre-mature loss of the Pol-II–snU1RNP interaction. In the latter case, the exon–intron junctions might be post-transcriptionally determined via exon definition. Remarkably, this is the process that is in general studied in in vitro splicing reactions, wherein the splicing process is uncoupled from mRNA transcription. There are reported differences in the requirement on E-complex factors between in vivo and in vitro studies. Those differences were mainly observed in the case of E-complex specific factors, such as SF1 and Prp40, which are not implicated in later steps of splicing. Interestingly, Prp40 has recently been shown to be specifically required for the inclusion of exons that are too small to harbor splicing enhancers, which are normally required in an exon-definition model of splicing [163]. The coupling of transcription and formation of the E-complex via Prp40 may therefore ensure intron definition in those cases in which exon definition is impossible.

## 15. Co-Transcriptional Formation of the E-Complex in Regulated and Alternative Splicing

The development of alternative splicing in higher eukaryotes stems from the ability to regulate splicing events in an intron-dependent fashion. This has been achieved during evolution by the parallel degeneration of splice sites and the increased complexity of the spliceosome. As described above, the comparison of the E-complex in *S. cerevisiae*, *S. pombe*, and mammals supports the development of a dedicated early spliceosomal complex to ensure splicing of introns with degenerated splice site motifs. This provides the opportunity to regulate splicing in an intron-dependent manner, which thereby would enable the development of alternative splicing.

In yeasts, regulation of splicing is mainly used to adapt to changes in the environment. An example would be nutrient starvation, which might also induce a change from mitotic growth to a meiotic cycle to form spores. While the vast majority of introns are constitutively spliced in *S. cerevisiae*, three introns have been reported to be exclusively spliced in meiosis [164]. Remarkably, in those introns, the otherwise conserved 5′ ss diverges from the consensus sequence and splicing of these introns requires Mer1, a protein exclusively expressed during meiosis. Mer1 enhances the recruitment of snU1 to the suboptimal 5′ splice sites, allowing the meiotic-specific splicing in budding yeast. In *S. pombe*, co-transcriptional splicing regulates the meiosis-specific intron excision and expression of an isoform of Rem1, a cyclin that is required for the meiotic cell cycle progression [165]. The co-transcriptional regulation of splicing is in this case achieved though promotor binding of two transcription factors. Mei4 induces transcription and splicing of Rem1, while Fkh2 is responsible for intron retention of the transcript during vegetative growth [166]. Beyond those two examples, intron retention appears to be the major form of splicing regulation in yeasts, and splicing and transcription are often co-regulated. It is interesting to note that a genome-wide analysis based on intron lariat sequencing in *S. pombe* revealed that the rate of aberrant splicing is inversely related to the expression level: highly expressed genes are less prone to erroneous splicing [167]. This observation correlates with our proposal: co-transcriptional assembly of the E-complex has to occur in a certain time window before the snU1-RNP eventually detaches from the pre-mRNA, and before the end of the transcript is reached.

The proposed model of co-transcriptional E-complex assembly serves to describe many but not all mechanisms of alternative splicing. Intron retention can be simply explained by detachment of the snU1-RNP before transcription reaches the 3′ ss of the intron. Intron retention is favored by week 5′ splice sites, slow transcription, or long introns—essentially as described in yeast. Beyond that, alternative selection of 3′ splice sites may depend on co-transcriptional E-complex formation. In this case, a strong 5′ ss-snU1 interaction would be required to choose the first (but weaker) 3′ ss in the case of slow transcription. On the contrary, high transcription rates may instead favor skipping of a weaker 3′ ss if a high-affinity 3′ ss site is reached sufficiently quickly. Exon skipping may also be explained by a strong interaction between the 5′ ss and snU1 coupled with high transcription rates, which may result in skipping of a weak 3′ ss and favor the binding to a strong 3′ ss at the end of the following intron; in this case, the exon and both flanking introns would be spliced out. Different from that, selection of an alternative 5′ ss might be rather favored by post-transcriptional mechanisms, as detachment of the snU1-RNP may result in displacement of the E-complex from the pre-mRNA during transcription. If co-transcriptional assembly of the E-complex cannot be achieved, the spliceosome must be assembled later, which in the case of long introns and short exons might depend on exon- rather than on intron definition. In summary, our simplified proposal for defining the exon–intron junctions by co-transcriptional assembly of the E-complex relies on: (1) the strength of the 5′ ss-snU1 interaction, (2) the time required for the transcription of the intron (dependent on intron length and transcription speed), and (3) the strength of the SF1/U2AF binding to the 3′ ss. In the ideal case of constitutive exons, this mechanism allows a fast assembly of the E-complex during transcription and an immediate splicing of the intron, when transcription is completed.

Beyond the proposed model, it is also important to point out that the regulation of alternative splicing in mammals is a highly sophisticated and combinatorial process which ultimately defines which two splice sites are ligated together. In addition to the features mentioned above, the RNA secondary structure, splicing regulatory elements, the presence of post-transcriptional nucleotide modifications, post-transcriptional modification of the trans acting factors, histone modifications regulating transcription, and many more factors might ultimately result in the highly extensive mechanism of alternative splicing, by which 20,000 protein coding genes translate into more than 90,000 different proteins in humans. Dissecting the impact of all the known factors on the outcome of a splicing reaction already represents an enormous challenge for the future. To understand the underlying molecular mechanisms, *S. pombe* might provide an excellent model organism for the future, as the E-complex in *S. pombe* and mammals is highly conserved and splice sites are similarly degenerated. Moreover, splicing appears to be regulated in *S. pombe* by phosphorylation and the kinases that carry out this function, such as Prp4 and Dsk1, are conserved in mammals [168,169,170,171]. Using a model organism such as fission yeast, in which many aspects of constitutive and regulated splicing are conserved, provides the opportunity to understand the basics of this processes, and the overall reduced complexity may help to dissect the mechanistic bases of alternative splicing.

## 16. Limitations and Future Perspectives

An ever-growing amount of data indicates that transcription and splicing are coordinated with each other in a cellular context. However, many studies on the mechanisms regulating splicing have been performed in vitro, wherein splicing is uncoupled from transcription. This might be one of the underlying causes for some of the discrepancies when comparing in vitro and in vivo experiments. For example, the rates of splicing are reported to be ten times faster in vivo and the requirement on certain splicing factors, particularly factors of the E-complex (such as U2AF35, SF1, Prp40) are different in studies in vitro and in vivo. In future work, effort should be devoted to establishing an in vitro transcription–splicing system, similar to the well-established in vitro transcription–translation reactions. Studies in a genetically traceable organism such as *S. pombe*, in which all factors of the mammalian E-complex are well conserved, might be an excellent alternative for an in vitro system.
